# Ecological limits: Science, justice, policy, and the good life

**DOI:** 10.1111/phc3.12740

**Published:** 2021-05-06

**Authors:** Fergus Green

**Affiliations:** ^1^ Department of Philosophy Utrecht University Utrecht Netherlands

## Abstract

Recent years have witnessed a revival of scientific, political and philosophical discourse concerning the notion of ecological limits. This article provides a conceptual overview of descriptive ecological limit claims—i.e. claims that there are real, biophysical limits—and reviews work in political and social philosophy in which such claims form the basis of proposals for *normative* limits. The latter are classified in terms of three broad types of normative theorising: *distributive justice*, *institutional/legal reform*, and *the good life*. Within these three categories, the article reviews normative proposals for limits on both aggregate‐level and individual‐level ecological exploitation. It also considers the relevance of political and ideological facts to the normative analysis of ecological limits, raising methodological questions about how normative theorists should respond to a world facing escalating ecological challenges.

## 1 INTRODUCTION

From claims of “peak oil” and climate “tipping points” to proposals for climate stabilisation goals and “planetary boundaries”, recent years have witnessed a revival of scientific and political discourse concerning the notion of ecological limits (Dobson 2016). The climate crisis and a plethora of other ecological concerns have prompted philosophers, too, to make various kinds of claims about ecological limits. Reviewing these claims, one is struck by their diversity. Ecological limits receive expression in terms of widely varying normative vocabularies, from theories of “natural resource justice” (Armstrong 2017) to “capability ceilings” (Holland 2008), from an “ethos of restraint” (Hayward 2009) to “personal carbon allowances” (Hyams 2009). The purpose of this article is to review and bring some order to this complex array of material, and to suggest some promising paths forward.

I classify ecological limit claims, at their broadest level of generality, along two dimensions. The first dimension concerns the *type of limit claim*, which I divide into two categories: *descriptive*; and *normative*. I subdivide the descriptive category into *resource limits* and *system limits*, and the normative category into *distributive justice*, *institutional/legal reform*, and *the good life*. The second dimension is the *level* at which the limit is posited (Spengler 2016, p. 927). For the purpose of this analysis, I divide this into two discrete categories: *individual‐level* and *aggregate level*, recognising that the latter encompasses a wide range of possibilities between the planetary level and a multiplicity of lower‐level collective units (e.g. national level, ecosystem level, etc.). These dimensions are represented in the headings of Table 1, below, with each cell populated with an example. I should emphasise that this is not the only way of carving up the terrain. In particular, the normative categories inevitably overlap somewhat. Nonetheless, I have tried to capture significant functional differences in the types of theorising that have been done on ecological limits. A possible third dimension, applicable to normative claims, is their ideal versus non‐ideal nature, understood here as their degree of “fact‐sensitivity”. I will touch on this issue where relevant in my discussion of normative work.

**TABLE 1:** Typology of ecological limit claims, with examples

Claim type Level	Descriptive		Normative		
	Resource limits	System limits	Distributive justice	Institutional/legal reform	The good life
Aggregate	Limited stocks of oil	Climate tipping points	Implied aggregate limits on natural resource use	Legislated national greenhouse gas emissions limits	Ethos of restraint; Anti‐fossil fuel norms
Individual	n/a	n/a	Capability ceilings; Functioning constraints	Legislated personal carbon allowances	Environmental virtues

Beyond its conceptual‐clarificatory function, this framework is used to structure this article. Part 2 discusses descriptive claims. These are claims about what the world is actually like, i.e. claims that there are real, *biophysical limits*. I review some recent prominent claims that there are biophysical limits, placing these in the context of historical discourse on environmental limits dating back to the 1970s. In light of this discussion, I identify and describe the two sub‐categories of biophysical limit claims mentioned above (resource limit and system limit claims), before discussing some key philosophical issues concerning the (contested) status of such claims, with an eye to their implications for normative theorising.

In Part 3, I review proposals for ecological limits in normative theorising, structured according to the above‐mentioned subcategories (distributive justice; institutional/legal reform; and the good life). There is a voluminous literature on normative theory concerning the environment. This review is limited to those works that specifically invoke the notion of upper limits on ecological exploitation (or similar). Maintaining this boundary‐line has proven easier with respect to theories of justice (section 3.1) and institutional/legal proposals (section 3.2) than is the case with regard to the more aretaic and teleological constructs discussed in section 3.3. Accordingly, section 3.3 is shorter and more synoptic than the other two sections in Part 3, and serves as more of a portal into wider conversations in environmental ethics than a review of specific proposals. Part 4 concludes with some suggested directions for future research.

## 2 DESCRIPTIVE CLAIMS ABOUT ECOLOGICAL LIMITS

### 2.1 Setting the scene: Some prominent claims about biophysical limits

The notion of ecological limits came to prominence in the 1970s following publication of the influential report by the Club of Rome, *The Limits to Growth* (Meadows et al., 1974). The report uses a computerised systems analysis methodology to model global development scenarios that capture interactions between variables relating to five “trends of global concern”: “accelerating industrialization, rapid population growth, widespread malnutrition, depletion of non‐renewable resources, and a deteriorating environment” (ibid 21). The authors first modelled a “business as usual” scenario up to the year 2100, finding the *depletion of non‐renewable resources* to be the feature that determined eventual system collapse. The standard sceptical response was that the stock of non‐renewable resources is likely to be larger, and used more efficiently, in the future than what was known at the time (e.g. due to improvements in science and technology) (see Dobson 2016, p. 290). In response, the authors doubled the assumed stock of resources. The model still projected economic collapse—albeit this time the determinative constraint was *environmental pollution* resulting from the additional economic production growth enabled by the larger assumed stock of natural resources (e.g. overuse of land causes erosion, which causes a decline in food production) (Meadows et al., 1974, p. 141).

*The Limits to Growth* was subjected to critique from various quarters (see Dobson 2016, pp. 291–96), which “were convincing enough to push the idea from center stage for much of the 1990s” (ibid 297). However, the notion of biophysical limits has returned to prominence in various forms in the 21^st^ century (ibid 297–301). It is instructive to consider perhaps the most influential contemporary variant of the notion of biophysical limits at the planetary scale: the *Planetary Boundaries framework*.

In a series of influential papers, Johan Rockström, Will Steffen and colleagues (Rockström et al. 2009a, 2009b; Steffen et al., 2015) develop the notion of “planetary boundaries” to guide human activities in coupled human–environmental systems with a view to ensuring that biophysical conditions remain conducive to human development in the way that they have during the Holocene era. The authors identify nine relevant systems and associated response variables: climate change; biosphere integrity (functional and genetic biodiversity); land‐system change; freshwater use; biochemical flows (phosphorous and nitrogen); ocean acidification; atmospheric aerosol loading; stratospheric ozone depletion; and “novel entities”^1^ (Steffen et al., 2015). Intrinsic to these systems, the authors posit, are *thresholds*: points at which some biophysical variable of interest (the “response variable”) undergoes a non‐linear transition in its functioning (Rockström et al., 2009b, p. 2). It is these thresholds that constitute descriptive ecological limit claims insofar as they posit the existence of a real biophysical phenomenon. By contrast, the *planetary boundaries* the authors define are human‐constructed limits to relevant “control variables” for each system, determined in relation to scientific knowledge about the relevant thresholds. The planetary boundaries are informed by normative judgements regarding matters such as what is an acceptable degree of risk to human development of crossing a threshold, given scientific uncertainty over the precise location of the threshold (Rockström et al., 2009b, pp. 3–5).^2^

Consider climate change as an example to illustrate the framework. The authors' proposed climate change boundary aims to avoid crossing thresholds that trigger “highly non‐linear, possibly abrupt and irreversible” changes in various response variables, such as the collapse of the thermohaline circulation^3^ (ibid 9). One of the two proposed control variables is the concentration of carbon dioxide (CO_2_) in the atmosphere, for which the authors suggest a planetary boundary of 350 parts per million (ppm), representing the lower bound of the zone of uncertainty regarding the location of the threshold (Rockström et al., 2009b, p. 10; Steffen et al., 2015, p. 2). The fact that CO_2_ levels are well in excess of the boundary (as of 2020 they exceeded 410ppm) is one of the principal concerns motivating contemporary scientific, political and philosophical discussions of ecological limits.

### 2.2 Two kinds of biophysical limit claims

In light of the foregoing discussion, we can distinguish two generic types of biophysical limit claims. The first type is a claim about *the finite availability of a natural resource stock or flow*. I call this type of claim a *resource limit claim*. A common example is a claim about the limited stock of a non‐renewable natural resource, like oil. Resource limit claims are more intuitive to understand, since they invoke a layperson's sense of what it is for something to be limited. Natural resources are part of larger ecological systems, the processes of which may replenish certain natural resources over timelines relevant to humans. The availability of such “replenishable”, or “renewable”, natural resources is thus time‐dependent. For example, there may be a limit to the amount of timber in a forest available for harvest *this year*. The availability of such resources is also *system‐dependent*. To continue the example, more timber will become available for harvest in a later year, *so long as the relevant ecosystem remains intact*.

The system‐dependence of natural resources provides one important motivation for protecting ecological systems: if a resource is overexploited or the relevant system otherwise excessively perturbed, its capacity to replenish natural resources may diminish or be destroyed. Other instrumental motivations for protecting ecological systems include the “regulating” services they provide, such as air and water purification and biodiversity maintenance, and their cultural and aesthetic value (Duraiappah 2004, pp. 13–14). These considerations bring us to the second type of biophysical limit claim, which is about *the finite capacity of an ecological system to withstand perturbations while remaining in its current state*. I call this type of claim a *system limit claim*. It is this second type of claim that is being made by the authors of the Planetary Boundaries studies. System limit claims are less intuitive than resource limit claims, as they invoke abstract concepts from the field of complex systems dynamics.

Both types of claims are integral to sustainability science, and it is important for philosophers who invoke biophysical limit claims to be clear about what is involved in each.

### 2.3 Philosophical contestation about biophysical limit claims

Being empirical claims, biophysical limit claims are contestable in ways that are of interest primarily to philosophers of science. Since this paper is ultimately interested in normative work on ecological limits, I will only briefly mention here two types of such contestation, noting their significance for normative work.

First, biophysical limit claims invite ordinary scientific scrutiny among the scientific community. Here, normative theorists should be aware of the (debates about the) role of contextual values in science (Douglas 2009; Elliott 2017). Values necessarily play a role in the science of biophysical limits. For example, values inform the determination of the qualitative state in which it is claimed that a system should be stabilised (i.e. the motivation for positing a system limit). This is clear in the Planetary Boundaries studies, where the authors assume that the relevant Earth systems should be stabilised in “a state conducive to human development” (Rockström et al., 2009b, p. 23). Additionally, contextual values enter into the delineation of the system itself, and the assessment of scientific hypotheses about where the relevant thresholds in a system lie. For example, contextual values influence decisions about how much evidence is needed to accept a scientific hypothesis about the location of a threshold, and in deciding how to determine confidence intervals/uncertainty bounds.^4^

Awareness of such contextual values is particularly important when scrutinising scientific claims within the environmental sciences because some of the claims of these sciences are especially contested among scientific experts. This contestation is due to the complexity of many of these sciences and the fact that direct experimental tests of their hypotheses are often out of reach (in principle, or for ethical or practical reasons) (Parker 2017, p. 27). Philosophers making normative claims in light of scientific claims about biophysical limits should take particular care to consider the values implicated in these claims.

Second, ecological limit claims (when combined with widely‐shared normative values) often motivate prescriptions for far‐reaching social and political transformation and/or clash with dominant ideologies and worldviews. Accordingly, they are frequently the subject of more overtly politicised—often organised and strategic—contestation that takes place outside (or at the public interface of) established scientific institutions and processes. Consider, for example, the decades long effort financed by fossil fuel corporations to mislead the public about climate science (e.g. Oreskes and Conway 2010; Supran and Oreskes 2017).

How should normative philosophers take account of this second type of contestation when it is levelled at biophysical limit claims? This, I suggest, depends on whether one is doing ideal or non‐ideal theory. Ideal theorists can, according to the tenets of the standard ideal‐theoretic method, permissibly abstract from such contestation.^5^ However, the more non‐ideal (in the sense of “fact‐sensitive”) one's theorising, the more such contestation becomes relevant to one's normative theorising. For example, I will suggest in section 3.2, below, that those making proposals for institutional reform or other kinds of real‐world action should take seriously the prevalence of strategic contestation, since it affects the epistemic and ideological context in which reform proposals will be entertained by citizens and political elites.

## 3 NORMATIVE THEORY AND ECOLOGICAL LIMITS

Let us now assume the truth of the following two biophysical limit claims: that there are biophysical limits; and that, as the Planetary Boundaries work and its underlying science suggests, many of these limits are close to being or have already been crossed. What follows from these (assumed, yet quite plausible) empirical facts for normative theorising?

### 3.1 Ecological limits and theories of distributive justice

Theorising about distributive justice has been a central concern of normative analytical political philosophy since the publication of Rawls' *A Theory of Justice* (1971). However, canonical distributive justice theorists in the liberal tradition such as Rawls and Dworkin have been criticised for failing adequately to take seriously the implications of biophysical limits for their theories (e.g. Bell 2017).

More recently, however, theorists of distributive justice have begun to take seriously the idea that an agent's ecological exploitation^6^ requires justification to a far greater extent than has traditionally been assumed in liberal theorising (Armstrong 2017; Bell 2017, p. 284; Caney 2016; Hayward 2017; Vanderheiden 2009).^7^ Such theorists have focused primarily on two issues: the imperative to respect (i.e. avoid breaching) aggregate ecological limits, including by developing justifications for particular limits, and; for a given aggregate limit, the principles for distributing rights and duties associated with the consumption of natural resources and the conservation of ecological systems (cf. Caney 2020, secs. 2–6; Hayward 2017, pp. 313–14).

Two broad directions in this literature can be observed. The first, and seemingly dominant approach, involves the more or less evolutionary development of dominant theories of justice through the more detailed specification of their implications for human–environment interactions. One recent example is Chris Armstrong's (2017) theory of justice and natural resources, which works out implications of a cosmopolitan egalitarian theory of justice for questions about both the distribution of limited natural resources and the allocation of burdens and advantages associated with ecosystem conservation. The climate justice literature can also plausibly be seen as an instance of this kind of theorising: there are debates over what justice requires by way of an aggregate limit on anthropogenic net greenhouse gas emissions; and debates over how the resulting “emissions budget” should be distributed (see Caney 2020). The latter debate has been approached, moreover, in the light of more and less “ideal” assumptions regarding, for example, levels of compliance and feasibility constraints (ibid).

The second direction, taken by a smaller group of theorists, has been to introduce novel *theoretical* constructs *into* theories of justice themselves on the purported basis that such innovations better account for facts about biophysical limits. I will here consider two such proposals, which may be called “limitarian” (Robeyns 2017, 2019).^8^

Breena Holland has proposed an innovation to Nussbaum's capability theory of justice. Nussbaum's theory posits 10 central capabilities, and Nussbaum argues that states should constitutionally protect each person's right to a minimum threshold level of each capability (Nussbaum 2000, 2006, 287–91, 2011, 33–36). Holland's argument is as follows:^9^

Because protecting the environmental preconditions of some capabilities can undermine the economic conditions that enable other capabilities, adequate protection of all capabilities will require establishing *capability ceilings* in addition to capability thresholds (Holland 2008, p. 416).

Holland builds on Nussbaum's example of driving (gas‐guzzling) SUVs:

to stop people driving SUVs is to limit the ways people can move freely from place to place, which is one component of Nussbaum's bodily integrity capability. Of course, the threshold level of this mobility component may not be so high that it includes being able to move freely from place to place in SUVs, and Nussbaum clearly would not see this extent of mobility as a fundamental entitlement. Yet that is precisely why a capability ceiling is needed (Ibid 417).

Peeters, Dirix and Sterckx, however, argue that Holland's proposal is redundant, since the existence of the minimum threshold ensures that environmentally‐damaging activities that deprive people of their minimum capabilities will not be permitted where those activities themselves go beyond one's minimum entitlements. All relevant parties to the debate agree that driving an SUV goes beyond one's minimum entitlements, so, assuming it threatens others' enjoyment of their minimum entitlements, it would need to be curtailed; the capability ceiling is not needed to generate this result (Peeters, Dirix, and Sterckx 2015, p. 379).^10^

These authors further critique Holland's proposal on the ground that it is “not *having a capability*, but rather *deriving functionings from it*” that relevantly harms the environment (2015, 381, emphasis in original). “In order to prevent illegitimate interference, which would reduce another person's well‐being”, they suggest, “it might therefore be necessary to constrain people's functionings” (ibid 381). Indeed, they go on to argue that “people's functioning *combinations* should be constrained as a whole—in terms of their aggregate appropriation of environmental assets” (ibid 382, emphasis in original).

However, this proposal, if it is to be understood as an innovation at the level of a *theory* of justice,^11^ seems to be vulnerable to an equivalent version of these authors' first objection to Holland. If it is truly the universal achievement of the minimum capability thresholds that matters for justice (ibid 381), then the “functioning constraint” seems redundant because the theory will already rule‐out above‐threshold actions that prevent others' attainment of the minimum threshold.

Still, perhaps the redundancy charge is too quick. For one thing, Nussbaum's capability theory is a *partial* theory of justice, concerned with bringing all persons up to the minimum capability thresholds (Nussbaum 2006, pp. 71, 75, 291–92, 2011, 36); Nussbaum says less about the distributive rule(s) that ought to apply above the minimum threshold (Nussbaum 2006, pp. 71, 75, 292–95, 2011, 40–42).^12^ On Nussbaum's theory, claims on social resources to secure *above*‐minimum capabilities clearly have a lower priority, but it is not clear how resources should be redistributed away from those who enjoy above‐minimum capabilities to those currently below the minimum threshold (e.g. who, among persons who enjoy more than their minimum entitlements, should have to give up their surplus resources first?). Upper limits (i.e. *maximum* thresholds^13^) can play a role in determining the redistributive patterns that apply when dealing with resources of agents who are above the minimum threshold (e.g. prioritising redistribution away from those who have resources above the maximum ahead of those whose resources lie in between the minimum and the maximum).^14^ This suggests one potential direction in which proposals for capability ceilings or functioning constraints could be developed.

An alternative direction—perhaps one closer to these authors' wider aims—is to conceptualise such upper limits not as novel theoretical elements in theories of justice, but rather as heuristics or tools in non‐ideal processes of policy deliberation and design. The idea would be that reasoning about alternative policy options could be enhanced by using capability ceilings or functioning constraints to conceptualise the limiting *effects* on persons of environmental policies that aim to control environmentally damaging behaviour directly (cf. Spengler 2016, p. 935).^15^

It is to proposals that fall squarely in the institutional/legal reform category to which I now turn.

### 3.2 Ecological limits and institutional/legal reform

So far, I have considered two possible routes from theories of justice to the conclusion that society should respect limits on ecological exploitation: one route applies existing candidate theories of justice to the assumed facts about biophysical limits; another route adds additional theoretical elements to existing theories of justice. Yet, the theories surveyed tell us little about the *institutional form* that such respect for limits should take. I will now consider two broad categories of proposals involving institutionalised (i.e. legal) ecological limits—aggregate‐level limits and individual‐level limits. Since it has been an especially active area of philosophical enquiry, I will focus on the example of climate change, i.e. limits on the exploitation of the biosphere's capacity to absorb greenhouse gases while respecting biophysical limits in the climate system.^16^

If we have reasons of justice (or otherwise) to respect certain limits on ecological exploitation, then it follows relatively uncontroversially that such limits should be institutionalised at an appropriate administrative level. At that level, an ecological limit will often be expressed as a goal (or objective or target). Consider, for example, the greenhouse gas mitigation targets that have been legislatively enacted by many national and subnational governments and the EU in order to address climate change (Iacobuta et al., 2018).^17^ Such targets are often included in “strategic” or “framework” climate laws that also establish governmental processes and institutions to facilitate achieving the targets and to determine administrative accountability for doing so (Averchenkova and Nachmany 2017; Averchenkova, Fankhauser and Finnegan 2021).^18^ To *actually* achieve a relevant target/goal, however, the relevant government will typically need to take (further) executive action and/or enact (further) legislation to incentivise private actors to change their behaviour. The goal, we might say, needs to be *operationalised* (cf. Vanderheiden 2008).

There is a tendency, in the literature on ecological limits, to assume that, given we have reasons of justice to limit aggregate ecological exploitation (perhaps enshrined in law as an aggregate goal), such limits should be operationalised *via individual (possibly tradeable* ^19^ *) quotas* on ecological exploitation, be they allocated to group agents such as corporations, natural persons, or both (see especially Hyams 2009; Vanderheiden 2018). However, this assumption is misplaced. Whether individual quotas are the best policy instrument with which to operationalise an aggregate ecological limit in fact depends on a wide range of factors (cf. Spengler 2016, pp. 927, 929, 2018).

Consider two such factors that are pertinent to the choice of policy instrument for addressing environmental problems (including respecting aggregate‐level limits). Both considerations militate against the use of individual‐level quotas in addressing climate change, but may militate in favour of quotas for addressing other environmental problems.

The first such consideration pertains to the *substitutability* of the harmful product or activity.^20^ Where the prospects for substitution (e.g. through technological innovation) are weak, then there may be a stronger case for (tradeable) quotas, since achieving the aggregate goal becomes a matter of legally limiting and distributing access to the relevant resource itself. However, if it is possible to respect an aggregate‐level limit by the invention and/or diffusion of substitutes for the environmentally harmful activity/product, then it may be preferable to enact policies that are geared toward the invention and mass diffusion of the substitutes. As leading climate ethicists have noted, a great deal of the greenhouse gas emissions that cause climate change are produced as a result of activities and technologies that are substitutable (e.g. Caney 2012, pp. 285–91; O'Neill, Holland and Light 2008). This being so, a better justified climate policy might focus on the innovation and diffusion of such substitutes. To replace fossil fuels in energy and industrial uses, for example, an effective combination of policies might include government‐funded research and development, subsidies for the demonstration and deployment of new technologies, government provision of necessary infrastructure, and taxes to promote behavioural shifts toward the substitute (Acemoglu et al., 2012; Aghion et al., 2014). A (tradeable) quota scheme would *not* likely provide the best incentives for such results (Aldred 2016; Pearse and Böhm 2014). Yet, tradeable quota systems (a.k.a. “emissions trading” schemes) have dominated debates about climate policy instruments among normative theorists (e.g. Caney and Hepburn 2011; Hyams 2009; Page 2013; Vanderheiden 2018).

The second consideration concerns the *politics of normative ideas*. Philosophers typically evaluate normative policy proposals for environmental problems in a manner that abstracts from the political and ideological context in which their proposals are directed. To be politically relevant, however, the philosophical analysis of public policy must take account of more contextual facts than is typical in ideal theory. As Jonathan Leader Maynard argues, this should include

reflecting on how a certain normative system or prescription will play out in the political thinking of real‐world actors—focusing … [on] the likely forms of reasoning, assumptions and attitudes such arguments and claims might encourage in actual political practice by citizens and elites (Leader Maynard 2017, p. 307).

With regard to climate change and other complex environmental problems, the ideological context includes strategic attempts by vested interests to: mislead the public about biophysical limits (see Part 2.3, above); frame climate mitigation laws as economic burdens on working families (MacNeil 2016); and frame responsibility for environmental problems as matters of personal consumer choice (Downey 2015, pp. 18–19; Turner 2014). Proponents of quota systems for mitigating climate change in general, and proponents of *personal* quotas^21^ in particular (Hyams 2009; Vanderheiden 2018), have largely ignored the very real danger that their proposals will play into the hands of such vested interests, potentially making it less likely that aggregate‐level ecological limits will be respected.

These considerations raise the question of how “fact‐sensitive” the philosophy of public policy should be, especially with respect to political and ideological facts. Certainly, institutional proposals for addressing climate change and other ecological limits vary widely in their stance on this issue. For example, suppose individual emissions quotas really are the “best” policy mechanism for tackling climate change, in some sense that abstracts from relevant political and ideological facts. The implication would then seem to be that we should *change the political conditions* that constrain its implementation. Simon Caney has proposed allocating “second‐order responsibilities” to effect such change in relation to climate action (Caney 2014, pts. IV–V).^22^ However, when it comes to climate change and many other ecological limits, time is of the essence, and Caney's proposal merely pushes the problems of motivation and collective action “up” one level, to the implementation of such “second‐order responsibilities”. Accordingly, Green and Brandstedt (2020) urge theorists to work with *already motivated* agents as part of a more politically “engaged” approach to climate ethics—one that takes political and ideological constraints and opportunities seriously when constructing normative ideas and policy proposals.

### 3.3 Ecological limits and the good life

Nonetheless, it seems clear that any institutional reform proposals sufficiently ambitious to respect biophysical limits, in the climate system and otherwise, will depend upon shifts in the political agency of many individuals and group agents, and hence in their motivating values and beliefs. Indeed, we seem to need to instantiate alternative visions of “the good life” to motivate political action, but also to change the more mundane personal, social and economic habits and practices that threaten ecological limits. This, at least, is an important impetus behind a wide range of work in normative theory that does not fall straightforwardly into theories of justice or specific policy proposals, but which is manifestly concerned, at least in part, with ecological limits and their implications.

For example, numerous scholars have found, in the recognition of ecological limits, the necessity of and inspiration for new environmental *virtues* and *vices* (Jamieson 2007, 2014; Sandler 2007; Sandler and Cafaro 2005; Wensveen 1999; Zwarthoed 2015), *practices* (Schlosberg and Coles 2016), moral‐social *norms* (Green 2018), and *ethos* (Butt 2017; Hayward 2009). *Values* such as freedom (Fragnière 2016; Lambacher 2016), autonomy (Vanderheiden 2009) and wellbeing (J. O'Neill 1993), as well as social, economic and institutional *practices of valuing* (J. O'Neill 1993; J. O'Neill, Holland, and Light 2008), have come in for critical reconceptualization in the light of ecological limits. In a similar vein, Melissa Lane (2011) draws on ancient philosophy to challenge contemporary inertia in the face of ecological crises and to provoke new forms of sociopolitical *imagination* and initiative. Indigenous and non‐western scholars have drawn attention to the rich resources in indigenous and non‐western philosophical traditions for rethinking our value‐orientations along more ecologically conscious lines (Whyte 2017; Whyte and Cuomo 2017; Winter 2020). Meanwhile, numerous political theorists have urged a reconceptualisation of fundamental *political institutions* along ecological lines (Dobson 2003; Eckersley 2004; Hayward 2006).

I have barely skimmed the surface of this work, which tackles environmental issues and themes beyond ecological limits per se and brings us onto the much wider fields of environmental ethics and green political theory.^23^ Let me conclude this section, then, with two observations. First, as with the work on distributive justice and institutional/legal reform, there is a similar division in ethically‐oriented work between more individually‐focused ethical constructs, like green virtues, and more structurally and collectively‐focused constructs, like social practices and *ethos*. However, individual‐level virtue‐ethical constructs are not *themselves* framed in terms of limits; rather, they are understood as behavioural dispositions conducive to maintaining aggregate‐level limits. This less direct form of individual contribution to respecting aggregate limits may avoid some of the problems raised by individual‐level limits discussed in the previous two sections.

Second, much of this work focuses on the ethical constructs it would ideally be good to instantiate. By contrast, a smaller but seemingly growing strand of normative theorising engages closely with agents who are already motivated and active in shaping the cultural‐ideational context, and thus centres contemporary opportunities and constraints in its approach to the (re)construction of values, norms, virtues, practices etc. (Green and Brandstedt 2020). For example, some theorists are leveraging the many interconnections between ecological themes and other issues that are of concern to already‐active cultural and social movements—those grounded in class/labour, racial, indigenous, feminist, anti‐colonial and other progressive projects—with a view to forging new ideological and political alignments capable of responding to interlinked challenges (Agyeman, Bullard, and Evans 2003; Bullard 1990; Green 2017; Hathaway 2020; Healy and Barry 2017; Prakash and Girgenti 2020; Schlosberg 2007; Walker 2011; Whyte 2017).

## 4 CONCLUSION

As ecological devastation and climate change continue apace, biophysical limits may well be one of the defining themes of the 21^st^ century. It is therefore a welcome development that normative theorists appear to be turning their attention in ever greater numbers to the consideration of normative limits on ecological exploitation. In this article, I have sought to classify and review ecological limit claims, focusing on descriptive claims about biophysical limits and normative work in which notions of biophysical limits play a central role. The normative discussion distinguished work in distributive justice from work focused on institutional/legal reforms and from a wider literature on values, practices, virtues and other ethical notions concerned with living well within ecological limits. The review sought to highlight some key debates within the literature to give a sense of the promise and pitfalls of theorising about ecological limits. I will conclude with some reflections on promising paths for future scholarship that, to my mind, are suggested from the preceding analysis, focusing on two themes that cut across the various sections in Part 3.

First, throughout the normative analysis, I cautioned against moving too quickly from the acceptance (in light of biophysical limit claims) of normative and institutionalised limits on *aggregate* ecological exploitation to the conclusion that *individual‐level* limits on ecological exploitation are justified—whether such individual limits take the form of new theoretical constructs within theories of distributive justice, institutionalised individual quota systems, or new environmental virtues. It is in the space that one traverses when making this move that I see the most pressing need for further (empirically informed) normative scholarship. Three lines of enquiry seem to me most promising in this regard. First, further work on novel theoretical constructs like capability ceilings and functioning constraints could helpfully elaborate the distinctive function of these constructs, explain their relationship to minimal (e.g. sufficientarian) thresholds, and justify them. Second, individual‐level quotas for various forms of ecological exploitation merit further exploration in the philosophy of public policy, with particular care given to justifying/critiquing the move from aggregate limits to individual‐level quotas given the potential availability of alternative policy instruments (with different normatively‐relevant features), especially those aimed at the innovation and diffusion of substitutes for environmentally harmful activities/products. Third, there would be value in further exploring both individual virtues/vices and (their relationship to) collective social structures aimed at motivating and mobilising individuals to take ecologically sustainable actions.

Second, running through all of these topics is a methodological issue that has resurfaced at various points throughout this review. The issue concerns the degree of sensitivity to facts—particularly political and ideological facts—that normative theorists should take into account when theorising about ecological limits. Given the time‐sensitivity of problems involving ecological limits, a shift in the balance of normative theorising toward a greater proportion of fact‐sensitive theorising may well be in order. Elegant ideal theories, after all, will be of little use on an uninhabitable Earth.

## ENDNOTES

^1^
Novel entities are defined as “new substances, new forms of existing substances, and modified life forms that have the potential for unwanted geophysical and/or biological effects” (Steffen et al., 2015, p. 7).

^2^
Because the identification of thresholds is subject to uncertainty, the authors propose planetary boundaries at the lower end of the identified zone of uncertainty (correctly acknowledging that this implies a normative—specifically, a conservative—approach to risk) (Rockström et al., 2009b, p. 473).

^3^
This process acts as a conveyor belt of warm surface water to the polar regions, and plays a key role in regulating the local climate in various parts of the world: see, e.g., <https://gpm.nasa.gov/education/videos/thermohaline‐circulation‐great‐ocean‐conveyor‐belt>.

^4^
See above, footnote 2.

^5^
The distinction between “abstraction” and “idealisation”, and the kinds of idealisations deemed permissible, have been discussed in O'Neill (1987), Robeyns (2008) and Valentini (2009). This kind of abstraction from facts of social and political life, and ideal theory more generally, is more vigorously contested by realist political theorists: for discussion, see Rossi and Sleat (2014).

^6^
For interesting conceptualisations of humans' environmental interactions in terms of “ecological space”, see Hayward (2017) and Vanderheiden (2009).

^7^
Two further axes of debate about theories of justice that have been catalysed by, or otherwise explored in the context of, biophysical limits have been the geographic and temporal *scope* of justice. I cannot explore these voluminous debates here.

^8^
Robeyns (2017, 2019) does not argue for ecological limits per se. Rather, she argues for an upper limit on *wealth*, with one justification for that limit being “unmet urgent needs”—including collective action problems concerning ecological deterioration—that require public financial resources to address. That said, Robeyns includes ecological limits within a broader limitarian theory (personal communication). As to whether limitarianism is best construed as a (partial) theory of justice, a mid‐level principle, or a policy proposal, see Timmer (Forthcoming‐a).

^9^
For an interesting discussion of the relationship between this proposal and aggregate‐level biophysical limits, see Holland (2014, 159–64).

^10^
Technically, this result would obtain only if sub‐minimum benefits have lexical priority over, or sufficient weighted priority to outweigh, the above‐minimum benefits. Where this is not the case, the ceiling may do independent normative work within the theory (and see also the text preceding footnote 14, below).

^11^
Various passages of the paper suggest that the authors *are* concerned with theories of justice (see, e.g., at pp. 377, 381). This impression is reinforced when the authors go on to discuss a proposed “operationalisation of functioning constraints” in the form of personal carbon allowances (at p. 382). If functioning constraints require institutional operationalisation, then they are not themselves institutional proposals, and must implicitly be proposed elements of a theory of justice.

^12^
The distributive rule that should apply above the minimum threshold is a subject on which sufficientarians disagree: for discussion, see Huseby (2019).

^13^
The conception of a “threshold” in this discussion is intended broadly to mean a discontinuity in the normative reasons that apply on either side of a specific distributive level. Accordingly, there can in theory be “*maximum* thresholds” (i.e. an “upper limit” or “ceiling” is a maximum threshold) as well as the more commonly discussed “minimum thresholds” (see further Timmer, Forthcoming‐b).

^14^
However, proponents of such multi‐threshold views must carefully specify the distributive rules that apply in the three relevant ranges (below the minimum, above the maximum, and in between the two thresholds) and the priority rules that are needed to resolve conflicts between them, such as when respecting the upper limit would leave insufficient resources to raise some persons up to the minimum (Timmer, Forthcoming‐b).

^15^
Holland comes close to such a suggestion when she describes institutional legal limits as “an indirect way of establishing capability ceilings” (2008, 417).

^16^
This way of expressing the limits encompasses both the emission of greenhouse gases into the atmosphere and the erosion of carbon “sinks” (e.g., cutting down trees).

^17^
There are also internationally‐determined limits in the climate realm, as expressed, for example, in the Kyoto Protocol to the United Nations Framework Convention on Climate Change and, more recently (but in a less precise way), in the Paris Agreement.

^18^
For further exploration of the normative foundations of climate legislation, see Green (2017).

^19^
The tradability or otherwise of quotas is an important design feature amendable to philosophical analysis, but is not germane to my arguments below.

^20^
The issue of substitutability is also central to the “growth” versus “degrowth” debate among environmental scholars (e.g. Hickel and Kallis 2020), which is also relevant to the question of institutional/legal reforms in light of ecological limits.

^21^
Under a system of personal carbon quotas, liability is imposed on natural persons for end‐use consumption, rather than on corporations upstream in the supply chains of carbon‐intensive products.

^22^
Caney's (2014) proposal is not explicitly tied to the enactment of an emissions trading scheme, though he elsewhere defends such schemes in a relatively idealised way (Caney and Hepburn 2011).

^23^
For a more thorough overview of these fields, see, respectively, the *Philosophy Compass* article by McShane (2009), and Gabrielson et al. (2016).
